# Complete Global Total Electron Content Map Dataset based on a Video Imputation Algorithm VISTA

**DOI:** 10.1038/s41597-023-02138-7

**Published:** 2023-04-25

**Authors:** Hu Sun, Yang Chen, Shasha Zou, Jiaen Ren, Yurui Chang, Zihan Wang, Anthea Coster

**Affiliations:** 1 Department of Statistics, University of Michigan, Ann Arbor, MI 48104 USA; 2 Department of Climate and Space Sciences and Engineering, University of Michigan, Ann Arbor, MI 48109 USA; 3 MIT Haystack Observatory, Westford, MA 01886 USA

**Keywords:** Space physics, Natural hazards

## Abstract

Ionospheric total electron content (TEC) derived from multi-frequency Global Navigation Satellite System (GNSS) signals and the relevant products have become one of the most utilized parameters in the space weather and ionospheric research community. However, there are a couple of challenges in using the global TEC map data including large data gaps over oceans and the potential of losing meso-scale ionospheric structures when applying traditional reconstruction and smoothing algorithms. In this paper, we describe and release a global TEC map database, constructed and completed based on the Madrigal TEC database with a novel video imputation algorithm called VISTA (Video Imputation with SoftImpute, Temporal smoothing and Auxiliary data). The complete TEC maps reveal important large-scale TEC structures and preserve the observed meso-scale structures. Basic ideas and the pipeline of the video imputation algorithm are introduced briefly, followed by discussions on the computational costs and fine tuning of the adopted algorithm. Discussions on potential usages of the complete TEC database are given, together with a concrete example of applying this database.

## Background & Summary

Space weather refers to the adverse impact of the highly varying solar and geomagnetic activities on the technological society. Extreme space weather can potentially lead to damages of critical infrastructure and disrupt our daily lives. The terrestrial ionosphere is dynamic and highly variable depending on multiple factors, i.e., solar, interplanetary and lower atmosphere conditions, as well as geographic locations. Eruptive solar events, such as coronal mass ejections (CMEs), have the greatest impact on short term but large scale ionospheric variability^1–9^. As one of the five major space weather threats in the National Space Weather Strategy and Action Plan, ionospheric disturbance could degrade or disrupt satellite navigation and communication systems as well as long-distance radio communication.

In the last decade, ionospheric total electron content (TEC) and the relevant products, such as the rate of TEC (ROT), the ROT index (ROTI), and differential TEC (ΔTEC), have become the most utilized parameters in the ionospheric research community^10–16^. TEC can be calculated using the differential delays of multiple transmitted frequencies from the Global Navigation Satellite System (GNSS) satellites, which are initially designed for the Positioning, Navigation and Timing (PNT) services. Plasmas within the ionosphere delay the electromagnetic wave propagation, producing the largest naturally occurring error source in the GNSS PNT services. To improve the PNT service accuracy, the ionospheric impact has to be removed, in particular for single frequency GNSS receivers. For example, the Wide Area Augmentation System (WAAS) developed by the Federal Aviation Agency (FAA) estimates and removes the ionospheric TEC effect to improve the accuracy, integrity, and availability of the GPS PNT service. WAAS provides horizontal and vertical navigation for approach operations for all users at available locations. Variability in ionospheric TEC also impacts GNSS timing. Specification and forecasting the ionospheric TEC and its variability are of critical importance to our modern technological society.

Despite the wide usage of TEC in the ionosphere and space weather community, there are a couple of challenges in using the global TEC map data including large data gaps over oceans and potentially losing meso-scale ionospheric structures when applying typical reconstruction and smoothing algorithms, such as Spherical Harmonics fitting. We have introduced a video imputation algorithm^17^, *Video Imputation with SoftImpute, Temporal smoothing and Auxiliary data (VISTA)*, targeted at providing reliable and complete TEC maps based on partially observed maps, such as the madrigal TEC database^18^. We have demonstrated^17^ that the imputed maps show strong alignment with observed entries, reveal desired global patterns, and, at the same time, preserve the observed meso-scale structures that alternative algorithms often can not capture, especially matrix completion related methods which often fail at recovering local structures of maps like the TEC map. Other attempts have also been used to reveal the meso-scale structures, such as tomographic-kriging combined technique^19^. In this paper, we aim at describing and releasing the complete TEC map database, covering the period from 2005 to 2020, that we construct based on the video imputation method VISTA^17^. We apply the VISTA algorithm on the Madrigal gridded TEC data product^10,20^. The complete TEC map database can be used for various ionospheric physics and space weather applications, as we demonstrate at the end of this paper, based on our latest applied research^21^.

## Methods

### Video completion algorithm: a brief introduction to VISTA

The algorithm used for generating complete TEC maps is the VISTA method^17^, which is based on matrix completion theory^22–25^ and is extended to account for various complexities in TEC observations. It is capable of processing time series of matrix data with missing values: with an output of a complete series of the time sequence of matrices by filling in reasonable values in the missing entries. The TEC data have such a data structure - that of being a time series of matrices with a significant fraction of missing values, such as large patches of missing TEC in the oceanic areas because of the lack of observations in the absence of ground-based receivers, and scattered & non-systematic missing data on land.

Mathematically, the TEC maps over an extended time period (e.g. one day) can be represented by a sequence of *m* × *n* matrices {*X*_*t*_, *t* = 1, 2, …,*T*}, each of which has missing values and the locations of the missingness vary across different time points. For any arbitrary matrix *X*, let Ω denote the observed entries in *X*; i.e. Ω = {(*i, j*):*X*_*ij*_ is observed}. Following the notation^23^, the projection P_Ω_(*X*) is an *m* × *n* matrix keeping all observed entries of *X* and replacing all missing entries with 0. The objective of the VISTA method is to use the observed entries in the sequence of matrices {*X*_*t*_, *t* = 1, 2, …,*T*} to obtain fully imputed matrices. The basic structure of the VISTA method adopts a matrix factorization approach, i.e., seeking for matrices *A*_*t*_ and *B*_*t*_ such that ${A}_{t}{B}_{t}^{T}$ fills all missing entries in *X*_*t*_ while preserving the observed entries in *X*_*t*_ as much as possible. The spatial continuity, temporal smoothness and any other auxiliary information can be taken into account in the VISTA method, which makes it a flexible and powerful video imputation method for TEC maps.

More formally, for each of the *T* maps, the complete version is denoted as ${A}_{t}{B}_{t}^{T}$, with *A*_*t*_ being an *m* × *r* matrix and *B*_*t*_ being an *n* × *r* matrix. Here *r* is a pre-specified rank parameter, and we fix *r* = 181 for this project, which is the maximum possible rank of any individual TEC map. To estimate *A*_1:*T*_, *B*_1:*T*_, the VISTA method aims at solving the following optimization problem:

1 $$\begin{array}{c}\mathop{{\rm{\min }}}\limits_{{A}_{1:T},{B}_{1:T}}\left\{F({A}_{1:T},{B}_{1:T})\mathop{\Delta }\limits_{=}\frac{1}{2}\mathop{\sum }\limits_{t=1}^{T}{\parallel {P}_{{\Omega }_{t}}({X}_{t}-{A}_{t}{B}_{t}^{T})\parallel }_{F}^{2}+\frac{{\lambda }_{1}}{2}\mathop{\sum }\limits_{t=1}^{T}({\parallel {A}_{t}\parallel }_{F}^{2}+{\parallel {B}_{t}\parallel }_{F}^{2})\right.\\ \left.+\frac{{\lambda }_{2}}{2}\mathop{\sum }\limits_{t=2}^{T}{\parallel {A}_{t}{B}_{t}^{T}-{A}_{t-1}{B}_{t-1}^{T}\parallel }_{F}^{2}+\frac{{\lambda }_{3}}{2}\mathop{\sum }\limits_{t=1}^{T}{\parallel {Y}_{t}-{A}_{t}{B}_{t}^{T}\parallel }_{F}^{2}\right\},\end{array}$$

where *λ*_1_, *λ*_2_, *λ*_3_ are tuning parameters; and *Y*_1_, *Y*_2_,…,*Y*_*T*_ are *m* × *n* auxiliary data with no missing values, ${P}_{{\Omega }_{t}}\left({X}_{t}-{A}_{t}{B}_{t}^{T}\right)$ is the matrix where the residuals of all the missing entries of ${X}_{t}-{A}_{t}{B}_{t}^{T}$ are set to 0, and ||.||_*F*_ is the matrix Frobenius norm. We refer our readers to the algorithm paper^17^ for more explanations. In this work, the auxiliary data *Y*_t_ is obtained by applying the spherical harmonics^26^ fitting over *X*_*t*_, independently for all *t*. Spherical harmonics fitting with a lower order typically results in overly smoothing the data thus not complying with the observations as desired, but they can provide a coarse imputation with great spatial smoothness; on the contrast, spherical harmonics fitting with a high order may create artificial, undesirable structures in the imputed maps. The VISTA model takes advantage of the auxiliary data to learn the global, smooth structure of TEC maps, while not losing local information via other terms; and the learning rate of this global structure from the spherical harmonics fitting is controlled by tuning parameter *λ*_3_. The other two tuning parameters *λ*_1_, *λ*_2_ control the rank of the imputed map and the temporal smoothness of the imputed map, respectively. The larger the *λ*_1_ value is, the lower the rank of the imputed map, which leads to fewer local and global features captured. The larger the *λ*_2_ value is, the more temporal consistency is demonstrated in the imputed video. Proper choices of *λ*_1_, *λ*_2_, *λ*_3_ can avoid over-fitting and improve the temporal and spatial smoothness of the imputed TEC time-series.

The optimization problem in (1) is solved via updating the matrices iteratively till convergence, in the order of: *A*_1_→*A*_2_→…→*A*_*T*_→*B*_1_→*B*_2_→…→*B*_*T*_→*A*_1_→*A*_2_→… Each time one matrix is updated, with all other matrices fixed. At iteration *k*, the matrix updating rule for *A*_1_, *A*_2_,…,*A*_*T*_ is given by

2 $${A}_{t}^{(k+1)}={\left[\left(1+{\lambda }_{2}\left({{\bf{I}}}_{\{t < T\}}+{{\bf{I}}}_{\{t > 1\}}\right)+{\lambda }_{3}\right){\left({B}_{t}^{(k)}\right)}^{T}{B}_{t}^{(k)}+{\lambda }_{1}{\rm{I}}\right]}^{-1}{Z}_{t}^{(k)}{B}_{t}^{(k)},$$

where ${Z}_{t}^{(k)}$ is defined as:

3 $${Z}_{t}^{(k)}={P}_{{\Omega }_{t}}({X}_{t})+{P}_{{\Omega }_{t}^{\perp }}\left({A}_{t}^{(k)}{\left({B}_{t}^{(k)}\right)}^{T}\right)+{\lambda }_{2}\left({{\bf{I}}}_{\{t > 1\}}{A}_{t-1}^{(k+1)}{\left({B}_{t-1}^{(k)}\right)}^{T}+{{\bf{I}}}_{\{t < T\}}{A}_{t+1}^{(k)}{\left({B}_{t+1}^{(k)}\right)}^{T}\right)+{\lambda }_{3}{Y}_{t}.$$

Similarly, the updating rule for *B*_1_, *B*_2_,…,*B*_*T*_ is given by

4 $${B}_{t}^{(k+1)}={\left[\left(1+{\lambda }_{2}\left({{\bf{I}}}_{\{t < T\}}+{{\bf{I}}}_{\{t > 1\}}\right)+{\lambda }_{3}\right){\left({A}_{t}^{(k+1)}\right)}^{T}{A}_{t}^{(k+1)}+{\lambda }_{1}{\rm{I}}\right]}^{-1}{\left({Z}_{t}^{\left(k+\frac{1}{2}\right)}\right)}^{T}{A}_{t}^{(k+1)},$$

where ${Z}_{t}^{\left(k+\frac{1}{2}\right)}$ is defined as:

5 $${Z}_{t}^{\left(k+\frac{1}{2}\right)}={P}_{{\Omega }_{t}}({X}_{t})+{P}_{{\Omega }_{t}^{\perp }}({A}_{t}^{(k+1)}{({B}_{t}^{(k)})}^{T})+{\lambda }_{2}({{\bf{I}}}_{\{t > 1\}}{A}_{t-1}^{(k+1)}{({B}_{t-1}^{(k+1)})}^{T}+{{\bf{I}}}_{\{t < T\}}{A}_{t+1}^{(k+1)}{({B}_{t+1}^{(k)})}^{T})+{\lambda }_{3}{Y}_{t}.$$

The algorithm terminates when $\sum _{t}\left({\left\Vert {A}_{t}^{(k)}-{A}_{t}^{(k-1)}\right\Vert }_{F}^{2}+{\left\Vert {B}_{t}^{(k)}-{B}_{t}^{(k-1)}\right\Vert }_{F}^{2}\right)$ is smaller than a pre-specified threshold. The output of the model gives the imputation of *X*_*t*_ as ${\widehat{A}}_{t}{\widehat{B}}_{t}^{T}$, where ${\widehat{A}}_{t},{\widehat{B}}_{t}$ are the final estimators output by the VISTA method.

Based on the descriptions of the algorithm, we have the following tuning parameters for the VISTA method, see Table 1, which are stored as part of the header data of the released dataset.

**Table 1:** Description of tuning parameters of the VISTA method.

Category	Notation	Description
VISTA	*λ*_1_	control soft penalty on *A*_1:*T*_, *B*_1:*T*_ norms for sparsity of imputed maps
	*λ*_2_	control temporal smoothness of the imputed maps
	*λ*_3_	control learning rate from the auxiliary data
Auxiliary Data	*l*_*max*_	maximum order of spherical harmonics basis function
	*v*	control penalty on the spherical harmonics coefficients for sparsity

All of the parameters are included as metadata in each data file of the database. Parameters could differ from year to year, see details below for how we choose the parameter for each day of the database.

### Data pipeline

**Fig. 1:**
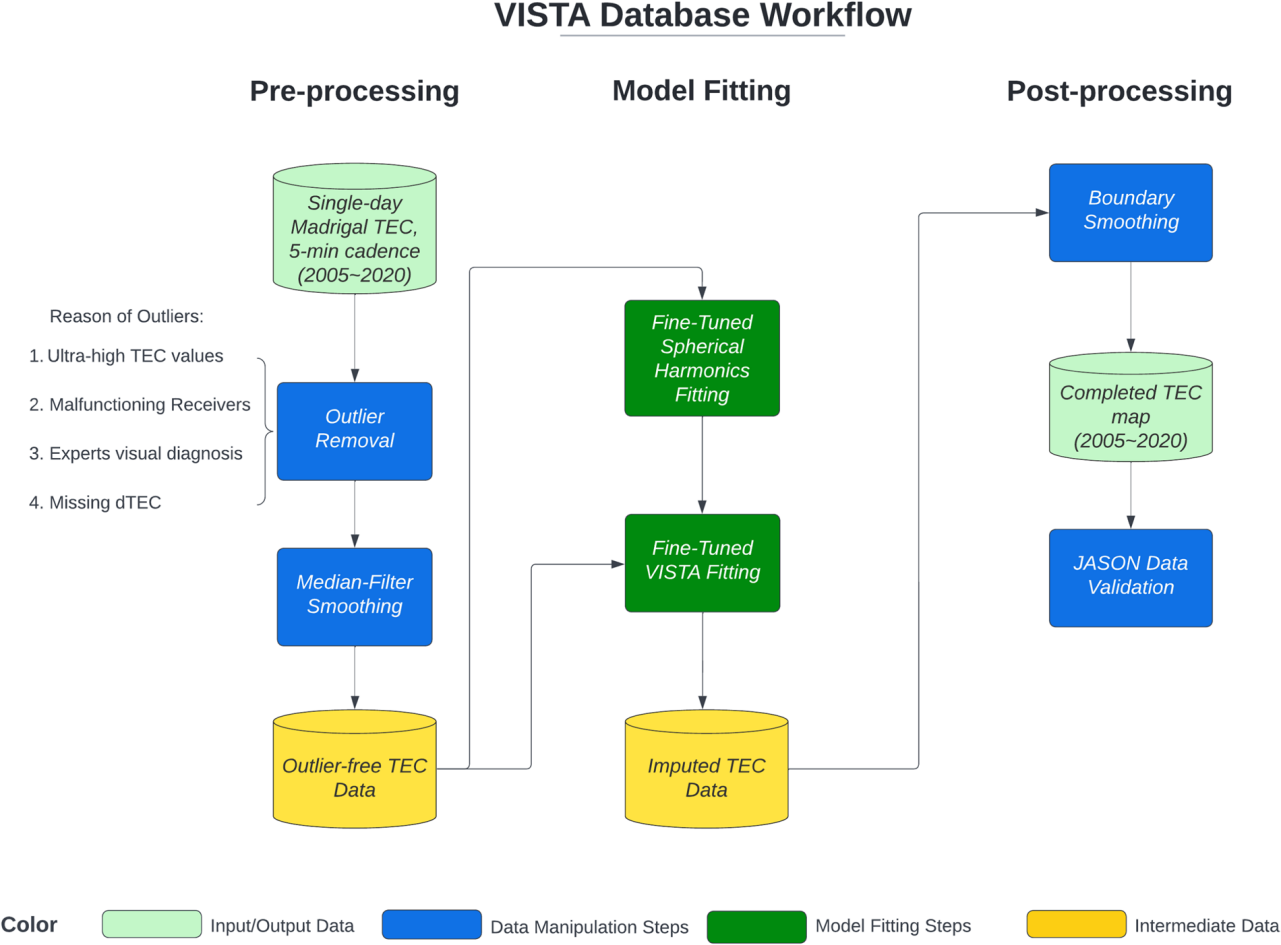
Complete Data Generating Workflow. The source data is the Madrigal TEC data containing missing values. We fit the spherical harmonics smoothing algorithm with *L*_2_ regularization to the source data, after removing outliers, to generate the auxiliary data. Combining both the source and the auxiliary data, we run the VISTA algorithm to generate the complete, low-rank and locally smoothed TEC map (the imputed TEC data). Finally, we run a moving average smoother to smooth the completed TEC maps near the day-to-day boundary to remove the impact introduced by daily fluctuations. More details on the VISTA fitting are included in Fig. 5.

In Fig. 1, we illustrate the full data processing procedures. All the data operations are put into one of the three categories: pre-processing, model fitting and post-processing. To summarize the workflow, we first pre-process the Madrigal TEC data by removing potential outliers. Then the outlier-removed TEC data are propagated into the Spherical Harmonics and VISTA algorithm in succession. Finally, we correct the inter-day biases by smoothing the imputed TEC maps near the day-to-day boundary and validate the database against an independent source of TEC measurements from the JASON satellite.

The whole framework chart shows a workflow that one may follow if one aims to generate complete TEC maps with new input data or apply the VISTA algorithm to generate other datasets. Note that one can replace the full block of Spherical Harmonics fit with other auxiliary data generating algorithm, or any existing auxiliary data, such as Global Ionosphere Maps(GIM) provided by International GNSS service (IGS) centers. We will briefly introduce the technical details of each of the three categories of data operations in the workflow below.

### Pre-processing: outlier removal

Before applying the Spherical Harmonics and the VISTA algorithm on the Madrigal TEC data, we first check the quality of the Madrigal data itself by removing data points that are considered as outliers. Four types of outliers are considered to remove, as detailed in Table 2. In general, the data availability and data quality of the Madrigal TEC improves as time approaches 2020, see panel (a) of Fig. 4. In the following three subsections, we describe the details of the first three types of outliers. The fourth type of outlier is based on setting up scientifically reasonable thresholds, and the order of which these outliers are removed follows: Others (1) and (2) → Unrealistically High-valued TEC → Distribution-based → Region-based → Others (3).

**Table 2:** Four Types of Madrigal TEC Data Outliers.

Outlier Type	Description
Unrealistically High-valued TEC (Type I)	TEC values from specific ground-based receivers whose daily TEC records are abnormal, e.g. have very high TEC values on record consistently during the night time (22LT ~ 6LT)
Distribution-based (Type II)	TEC values in the right tail of the daily TEC distribution (>95%), identified by fitting a kernel density estimator for the daily TEC distribution and check if a peak exists in the right tail.
Region-based (Type III)	TEC values within questionable geographic regions with either very low or very high TEC values compared to the neighborhood regions, e.g. unreasonably high TEC values in the Antarctica
Others	1) TEC value greater than 500 TECu; 2) without corresponding dTEC values; 3) dTEC ≥50 TECu and TEC ≥10 TECu and no more than one pixel with dTEC <50 TECu exists in the 3 × 3 neighborhood.

We recommend our users to read the user manual of the database on outlier removal for more details. Generally speaking, each frame of TEC map (181 × 361) have 0 ~ 5 outliers, and sometimes, though rarely, this amount can be around 100 ~ 200. The order of which these outliers are removed is: Others 1) and 2) → Unrealistically High-valued TEC → Distribution-based → Region-based → Others 3). dTEC is defined as the error in vertically integrated electron density and is measured in TECu.

#### Type I: Unrealistically high TEC values

**Definition:** Any location (identified by latitude-longitude) that observes unrealistically high TEC values, during 22 local time (LT) to 6LT, at a frequency that is above a pre-specified frequency threshold. We remove the TEC data at this location throughout the whole day, as these locations are suspected to have problems with its ground-based receivers during these days.

**Fig. 2:**
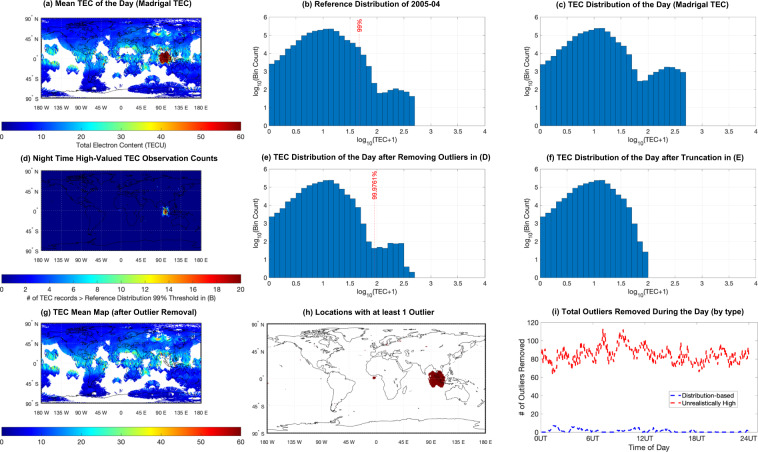
Example of unrealistically high-valued TEC outliers and distribution-based outliers for Apr 28th, 2005. Panel (**a**) shows the location-wise average TEC value of this day, where there is a large cluster of high TEC locations. Panel (**b**) shows the distribution of the TEC value for the whole month of April 2005, after subsampling the data points of each day at a ratio inversely proportional to the number of days in the month (i.e. 30 in this case). Panel (**c**) shows the distribution of TEC values before outlier removal for this day. Panel (**d**) shows the counts of TEC observations at each location that surpasses the 99% threshold (red line in (**b**)) of the monthly TEC distribution in (**b**), during the night time (22LT ~ 6LT). One can see that the high-TEC region is highlighted. Panel (**e**) shows the TEC distribution after removing these unrealistically high TEC values, and Panel (**f**) shows the same distribution with distribution-based outliers removed. Panel (**g**) plots the daily average TEC value, for each location, after removing the two types of outliers, and (**h**) highlights locations with at least one outlier removed. Panel (**i**) finally shows a breakdown of the number of outliers removed throughout the day.

An illustration of this type of outlier is shown in Fig. 2 for Apr 28th, 2005. Panels (c) and (e) show the change of the distribution of TEC values before and after outlier removal. To detect this type of outlier, we go through the following steps:

1. *Generating a reference distribution* (Panel (b)): we subsample daily TEC data within each month inversely proportional to the number of days per month, i.e. the longer the month, the lower the proportion. With the large sample size of the TEC data, subsampling only speeds up computation and reduces storage demand, without sacrificing accuracy on the estimation of quantiles.
2. *Thresholding* (Panel (d)): for each location (latitude × longitude), we count the number of TEC values during the night time (22LT ~ 6LT) that surpasses the 99% threshold of the reference TEC distribution (red vertical line in Panel (b)). Denote this count as *N*_*lat*, *lon*_. We then divide this count by the total number of night time observations for the location, denoted as *M*_*lat, lon*_. We classify this location as an outlier if: $\frac{{N}_{lat,lon}}{{M}_{lat,lon}} > \frac{3}{16}$ (the threshold is determined empirically).
3. *Dilating*: we generalize the outliers to locations near the detected locations in step 2. All locations whose 5 × 5 neighborhood contains at least 3 locations that are outliers under step 2 are classified as outliers. Then we remove all TEC observations of all such locations throughout the whole day, since these locations are likely to be associated with ground-based receivers that are unreliable during this day.

#### Type II: Distribution-based Outliers

**Definition:** Any data point that has a TEC value at the right tail (>95%) of the daily TEC distribution, and those whose TEC values belong to a peak in the fitted kernel density curve of the TEC distribution that is not the main peak of the TEC distribution. Equivalently, we assume that the daily TEC distribution is uni-modal, and any modes other than the major mode in the right tail are considered outliers. Thus we truncate the TEC distribution in the right tail to avoid having multiple modes.

Illustration of this outlier removal for Apr 28th, 2005 is shown in Fig. 2, where the change of the TEC distribution before and after the removal are reflected by Panels (e) and (f). The following steps detect this type of outlier:

1. *Picking Threshold* (Panel (e)): after removing outliers classified as “Others (1)”, “Others (2)” and “Unrealistically High-valued TEC”, we generate the TEC distribution of the day, and fit a kernel density curve. It is more common for any daily TEC distribution to follow a uni-modal distribution, so we pick the threshold that splits the first mode from any additional modes in the right tail (>95%).
2. *Thresholding* (Panel (f)): we apply the identified threshold to all data points remaining in the Madrigal TEC data, and remove any data points beyond the threshold. See Panel (i) on how many distribution-based outliers are removed frame-by-frame throughout the day. Daily plot has been randomly selected to perform visual checks to make sure the outliers identified in this way are randomly scattered and not part of the ionospheric high density structures.

#### Type III: Region-based outliers

**Definition:** Additional questionable regions identified with domain knowledge and visual assessment, especially in the Antarctica region. Persistently high or low TEC values comparing with the surrounding region despite of large scale background TEC changes are potentially questionable data. When a region is spotted as questionable, we remove its TEC records throughout the day. Those regions that are mis-classified as outliers, such as peaks of equatorial ionization anomaly(EIAs), are added back after careful inspection.

**Fig. 3:**
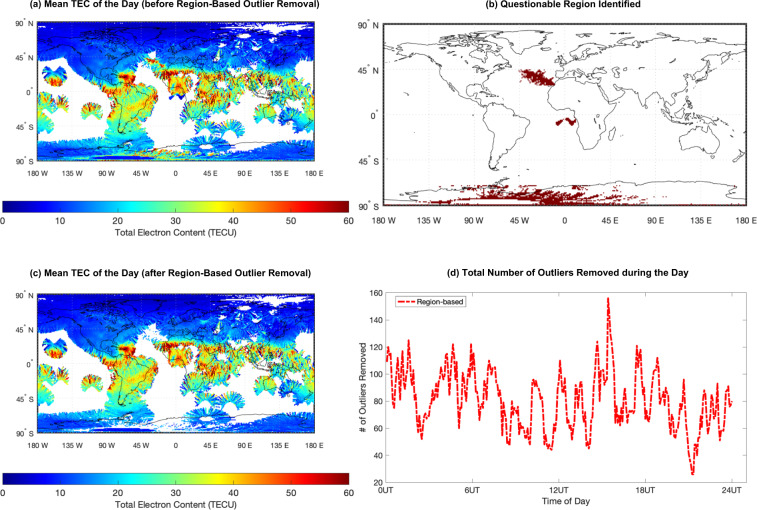
Example of the region-based outliers for Nov 26th, 2011. Panel (**a**) and (**c**) shows the location-wise average TEC value of the day, before and after the outlier removal. Panel (**b**) shows the locations that we consider as questionable and we remove the TEC records at these locations entirely throughout the whole day. Panel (**d**) tracks the number of data points removed for each frame within the day.

Illustration of this type of outlier for Nov 26th, 2011 is shown in Fig. 3, Panels (a) and (c) show the change of TEC maps before and after the removal. Since this type of outlier is identified manually, we are very cautious and only remove when we have full confidence. This type of outlier is the rarest among the four types and only appears on 2 days in 2005, 4 days in 2010 and 93 days in 2011.

#### Summary of outlier removal

In Fig. 2, we show one of the examples on April 28th, 2005, where outliers of the Madrigal TEC are evident. In panels (a) and (g), we show the daily average of TEC values for each location on the 1-degree latitude-longitude grid, before and after outlier removal. Panel (h) highlights the location where at least 1 outlier of any type in Table 2 is found. The big red patch highlights a group of unrealistically high TEC values (Type I), and the scattered red points are locations where records of unreasonably high TEC values are observed (Type I & II). After the outlier removal, we apply a 3 × 3 median-filter to smooth the data. These median-filtered maps are then used for model fitting.

**Fig. 4:**
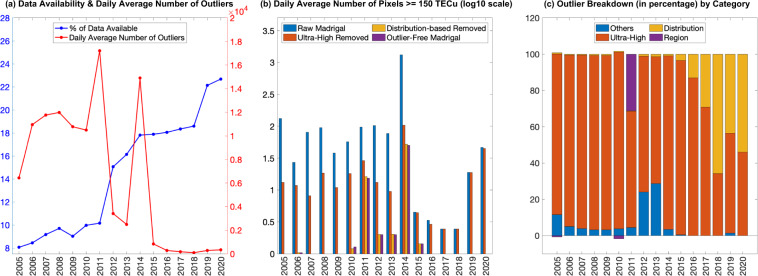
Summary of the outlier removal on the Madrigal TEC data. Panel (**a**) shows the data availability (%) in the raw Madrigal TEC data (blue) and the number of outliers removed per day across the 16 years period (red). Panel (**b**) shows the daily average number of pixels with at least 150 TECu, averaged at the year level, for four snapshots of the Madrigal TEC along the outlier removal workflow. Note that the y-axis is in log_10_ scale. Panel (**c**) shows the percentage of each of the four type of outliers removed, as defined in Table 2. In years 2005 and 2010, the percentage for region-based outlier is negative because on average, we add pixels that are mis-classified as outliers back to the data.

To give a holistic view of the amount of the four types of outliers in the Madrigal TEC data, we show a summary of the outlier removal step in Fig. 4. In the left panel, we show the yearly data availability (blue curve) in the raw Madrigal TEC (defined as the percentage of pixels with data out of all pixels in the Madrigal TEC before outlier removal), and the yearly average of the daily number of outliers removed (red curve). As one can see, the number of outliers is inversely correlated with the data availability and reduces significantly in more recent years. In the middle panel, we truncate the TEC distribution of each day and count the number of pixels with at least 150 TECu for four snapshots of the Madrigal TEC data along the outlier removal workflow. The bar plot is organized by year and show that after removing either the ultra-high TEC values (Type I) or the distribution-based outliers (Type II), the right tail of the TEC distribution (above 150 TECu) is greatly reduced. In the right panel, we show a yearly breakdown of the four types of outliers removed. Occasionally, the region-based outlier show a negative percentage, indicating that the mis-classified outliers are ingested back into the data after careful evaluation. Overall, there are more Type-I outliers that are likely related to problematic receivers before 2015, and more distribution-based outliers since 2015.

Outlier removal of the Madrigal TEC data is a challenging research problem on its own since no ground truth label indicating which pixels are outliers exists. The outlier removal steps taken to construct our database follow the principles that we are as conservative as possible and only remove pixels that violate even mild assumptions of the TEC distribution of the day. Furthermore, we want to emphasize that the VISTA algorithm is a robust algorithm. It is not sensitive to the presence of a few scattered outliers in the source data. There is minimal difference (<0.1 TECu overall) between the VISTA TEC map with a few pixels replaced by zero and the VISTA TEC map fitted from the Madrigal TEC map with the same set of pixels replaced by NaN (missing values). Thus, depending on the purpose of scientific applications, users can further screen out scattered outliers from our database if necessary without having to refit the VISTA model.

### Model Fitting: Standardization & Spherical Harmonics Algorithm

**Fig. 5:**
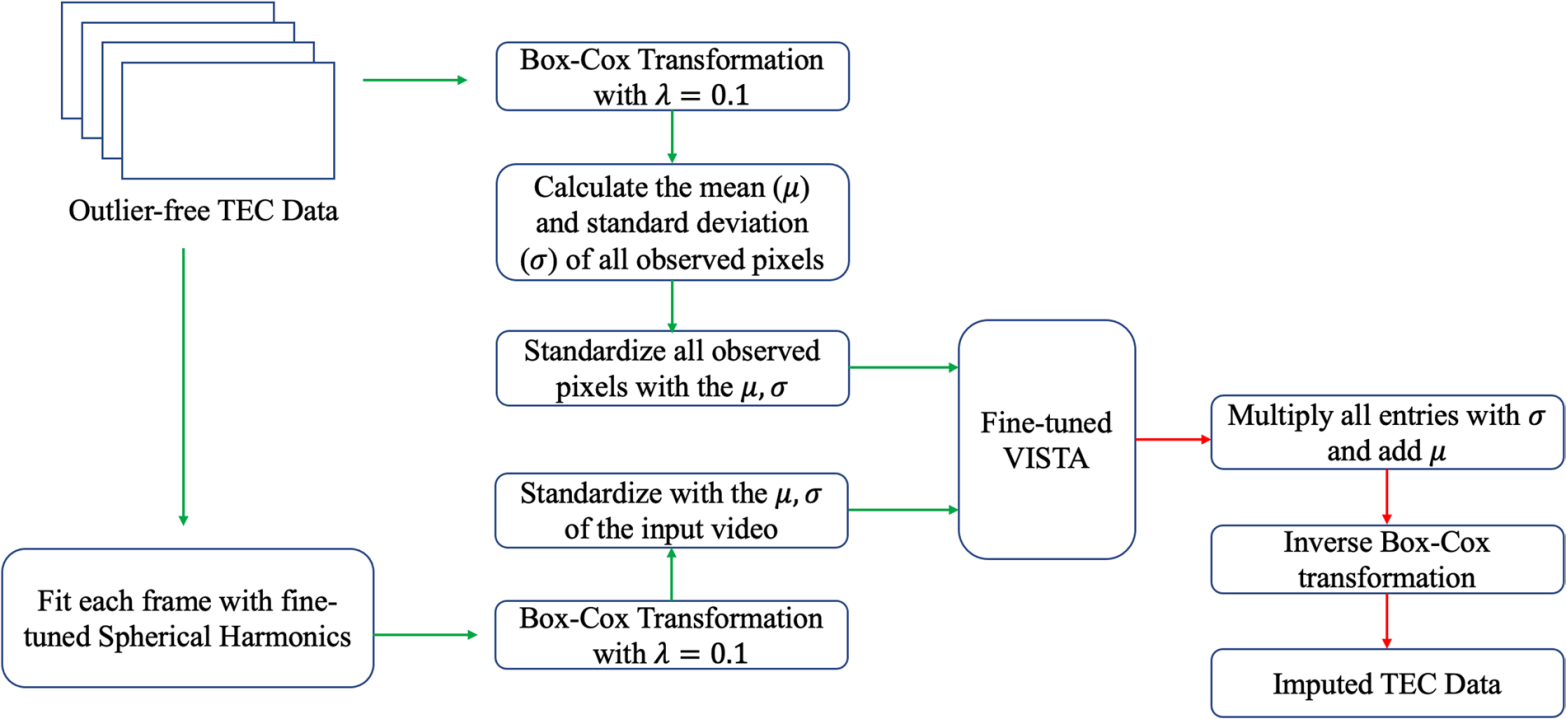
Detailed model fitting workflow. Starting with the input video (after pre-processing with outlier removal), we fit each frame with spherical harmonics to get a smoothed video. Then we use the Box-Cox transformation and normalization to pre-process the input and the smoothed videos, and feed both videos to the VISTA algorithm to generate an imputed video. Finally, we do reverse normalization and inverse Box-Cox transformation to convert the imputed video back to the original scale.

In an earlier subsection, we have briefly introduced the VISTA algorithm. In this subsection, we will introduce the rest of the details in the model fitting workflow, namely data standardization and spherical harmonics (SH) fitting. Figure 5 shows a more detailed version of the model fitting workflow in Fig. 1. The SH and VISTA fit requires a standardization step before the fitting and another step after the fitting to reverse the fitted values back to the original scales. The standardization contains a box-cox transformation step and a normalization step. The Box-Cox transformation^27^ is a method applied to each observed pixel of the input video (outlier-removed data) and every pixel of the auxiliary data (i.e. spherical harmonics fitted data) to make the data more like normally-distributed. Pixel-wisely, the Box-Cox transformation is doing ${y}^{{\prime} }=\frac{{y}^{\lambda }-1}{\lambda }$ for any pixel intensity *y*, for some tuning parameter *λ*. This could make the imputation more robust to extreme values. The normalization is making the pixel intensity distributed with mean 0 and standard deviation 1. Note that the mean and standard deviation used for normalizing the auxiliary data comes from the source data.

The Spherical Harmonics (SH) fitting creates a general estimation on the large-scale TEC distribution, which provides initial guesses over the oceanic areas that can facilitate the VISTA algorithm later on. Treating the TEC map at a given time *t* as a function on spherical coordinates, *X*_*t*_(*θ*, *ϕ*), we can approximate the TEC distribution using spherical harmonics expansion:

$${X}_{t}\left(\theta ,\phi \right)\approx \mathop{\sum }\limits_{l=0}^{{l}_{max}}\mathop{\sum }\limits_{m=-l}^{l}{a}_{l}^{m}{R}_{l}^{m}\left(\theta ,\phi \right),$$

where θ and ϕ are the elevation and azimuth angles. ${R}_{l}^{m}(\theta ,\phi )$ denotes a spherical harmonic basis with degree *m* and order *l* (|*m*| ≤ *l*), and ${a}_{l}^{m}$ is the corresponding coefficient. For convenience, we give each harmonic function with degree *m* and order *l* a unique index $j={l}^{2}+l+m+1$ and each observation an index *i* from 1 to *N*, then we have a system of linear equations in terms of the coefficients

$$\left[\begin{array}{ccc}{R}_{1}({\theta }_{1},{\phi }_{1}) & \ldots  & {R}_{k}({\theta }_{1},{\phi }_{1})\\ \vdots  & \ddots  & \vdots \\ {R}_{1}({\theta }_{N},{\phi }_{N}) & \ldots  & {R}_{k}({\theta }_{N},{\phi }_{N})\end{array}\right]\left[\begin{array}{c}{a}_{1}\\ \vdots \\ {a}_{k}\end{array}\right]\approx \left[\begin{array}{c}{X}_{t}({\theta }_{1},{\phi }_{1})\\ \vdots \\ {X}_{t}({\theta }_{N},{\phi }_{N})\end{array}\right]$$

that can be written as *RA* = *X*, where *k* = (*l*_*max*_ + 1)^2^ is the total number of harmonic functions and *N* is the total number of observations in the TEC map. Note that the X here is a column vector with its element being all the observed entries in *X*_*t*_. The coefficients $\widehat{A}$ can be obtained by solving a least-square optimization problem with Tikhonov regularization

$$\mathop{{\rm{\min }}}\limits_{A}{\left\Vert X-RA\right\Vert }_{2}^{2}+v{\left\Vert \Gamma A\right\Vert }_{2}^{2}$$

where Γ is a diagonal matrix with ${\Gamma }_{j,j}={l}_{j}({l}_{j}+1)$ and *l*_*j*_ denotes the order of the *j*^*th*^ harmonic function. The purpose of having Tikhonov regularization is to avoid overfitting artifacts by penalizing high-frequency harmonics. Lastly, negative TEC values are removed based on a method^28^ using inequality constraints. The output auxiliary map *Y*_*t*_ can be obtained by evaluating ${Y}_{t}\left(\theta ,\phi \right)=\mathop{\sum }\limits_{l=0}^{{l}_{max}}\mathop{\sum }\limits_{m=-l}^{l}{\widehat{a}}_{l}^{m}{R}_{l}^{m}\left(\theta ,\phi \right)$ at each latitude and longitude grid.

### Model fitting: parameter tuning

In our model fitting workflow, we label both SH and VISTA as “fine-tuned”. As listed in Table 1, VISTA requires 5 tuning parameters and we discuss the choices here. The rank parameter *r* is not tuned but is pre-determined as *r* = min(*m, n*), where *m, n* are the dimension for the input matrix *X*_*t*_. For the Madrigal TEC map, *m* = 181, *n* = 361, which corresponds to a 1 degree latitude by 1 degree longitude grid structure. The rest of the tuning parameters are determined sequentially in the order of: (*l*_*max*_, *v*), *λ*_3_, *λ*_2_, *λ*_1_. In other words, we first choose *l*_*max*_ and *v*, then *λ*_3_, and finally *λ*_2_ and *λ*_1_. It is desirable to choose all tuning parameters jointly, but considering the number of feasible combinations of all 5 parameters on fine grids and the computing time of VISTA, it is recommended that one chooses only a subset of tuning parameters at a time. In the next two subsections, we describe the parameter tuning for spherical harmonics fitting and the VISTA algorithm in details. See^17^ for demonstration on the parameter tuning with numerical examples.

Our final TEC map database covers the years from 2005 to 2020. We partition the 16-year period into four intervals: 2005 ~ 2011, 2012 ~ 2014, 2015 ~ 2018, 2019 ~ 2020. Within each interval, we pick one month of data to tune the parameters and consequently, all days within each interval share the same set of tuning parameters. The partition is chosen as such since these four intervals have relatively different data missing percentage in the raw Madrigal TEC data, where the missing percentage are >90% for 2005 ~ 2011, ~ 85% for 2012 ~ 2014, ~ 82% for 2015 ~ 2018 and <80% for 2019 ~ 2020. The four months are: 2009-Apr, 2014-Jan, 2015-Sep and 2019-May, which are chosen to cover different geomagnetic activity levels and different periods in the previous solar cycle. 2015-Sep is a stormy month with much higher activity level based on the geomagnetic Dst index^29^, which estimates the magnitude of the ring current in the inner magnetosphere. The other three months are non-stormy months in different phases of the solar cycle and different season, which makes the four months of data more representative of the whole Madrigal TEC database. The differences of the geomagnetic activity levels (stormy and non-stormy) would not affect the parameter tuning result, however, since all input data are scaled to have zero mean and unit variance before feeding into the algorithm (see Fig. 5).

#### Spherical harmonics tuning parameter

To tune *l*_*max*_ and *v*, we consider ${l}_{max}\in \{5,6,7,\ldots ,14,15\}$ and $v\in \{0.1,1\}$. For each frame of the TEC map, denoted as *X*_*t*_, we randomly “mask out” 20% of the observed pixels as the validation set and fit spherical harmonics with different (*l*_*max*_, *v*) combinations on the rest of the 80% observed pixels. We denote indices for the validation set for *X*_*t*_ as ${\Omega }_{t}^{\ast }$ and define the projection operator ${P}_{{\Omega }_{t}^{\ast }}$ similarly as in Section *Video Completion Algorithm: a Brief Introduction to VISTA*. With the fitted map, denoted as *Y*_*t*_, we calculate the rooted mean squared error (RMSE) of the fit on the validation set:

$${\mathrm{RMSE}}_{\mathrm{SH}}=\sqrt{\frac{{\parallel {P}_{{\Omega }_{t}^{\ast }}({Y}_{t}-{X}_{t})\parallel }_{F}^{2}}{{\parallel {\Omega }_{t}^{\ast }\parallel }_{0}}}$$

where ||.||_*F*_ is the Frobenius norm of a matrix and ||.||_0_ is the *L*_0_ norm of a matrix, i.e., counting the number of non-zero elements of a matrix. A similar RMSE is calculated for tuning the VISTA parameters *λ*_1_, *λ*_2_, *λ*_3_ below, where one can simply replace *Y*_*t*_ with the VISTA fitted map.

**Fig. 6:**
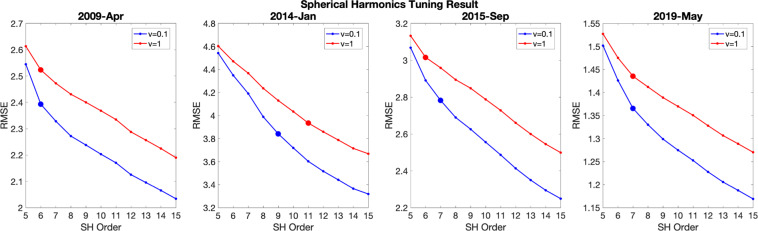
Spherical Harmonics Tuning Result: 2009-Apr, 2014-Jan, 2015-Sep, 2019-May. Red lines correspond to *ν* = 1 and blue lines correspond to *ν* = 0.1. Dots on the lines highlight the “elbow” of each error curve.

We report the validation set RMSE for each of the four months under all combinations of the tuning parameters (*l*_*max*_, *v*) in Fig. 6. We highlight the “elbow point” of each RMSE curve with a circle dot, which is the point that has a significant change of slope and is determined by sequentially bisecting the curve at each candidate point and fitting two linear models on the points to the left and right of the candidate point and choose the one with the lowest mean-squared error of the two fits. Based on the result of RMSE on the goodness-of-fit of spherical harmonics, we pick *v* = 0.1 for all years when generating the database and *l*_*max*_ = 6 for 2005~2011, *l*_*max*_ = 9 for 2012~2014 and *l*_*max*_ = 7 for the remaining years.

#### VISTA tuning parameter

**Fig. 7:**
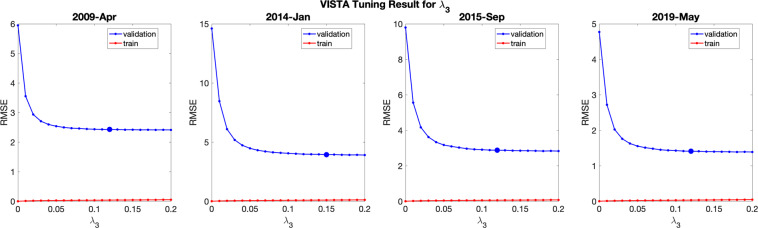
RMSE corresponding to *λ*_3_ tuning, during the tuning of which we fix *λ*_1_ = *λ*_2_ = 0. Blue line shows the RMSE on the validation set and red line shows the RMSE on the training set. Large blue dots show the *λ*_3_ that minimizes the average of the validation set RMSE and train set RMSE, which are the *λ*_3_ picked for each of the four months.

To tune parameters of the VISTA, we first tune *λ*_3_, with *λ*_2_, *λ*_1_ fixed at 0. The steps are very similar to the tuning procedure of *l*_*max*_ and *v*. We choose a grid of *λ*_3_, and “mask out” 20% of the observed pixels as validation set (same set as the SH tuning) and fit VISTA to get fitted maps. Finally we pick the best *λ*_3_ based on the RMSE. However, when tuning the parameters of VISTA, we pick the parameters that minimizes the average of the RMSE on the validation set and the training set to balance the quality of the imputation on the observed and unobserved pixels. Similar to the tuning procedure of *λ*_3_, we tune *λ*_2_ by fixing *λ*_3_ at its optimal values, and tune *λ*_1_ by fixing both *λ*_2_ and *λ*_3_ at their optimal values. The candidate sets are ${\lambda }_{1}\in \{0.1,0.2,\ldots ,1.9,2.0\}$ ${\lambda }_{2}\in \{0.00,0.05,\ldots ,0.95,1.00\},{\lambda }_{3}\in \{0.00,0.01,\ldots ,0.19,0.20\}$. Here we first show the RMSE results for *λ*_3_ in Fig. 7.

**Fig. 8:**
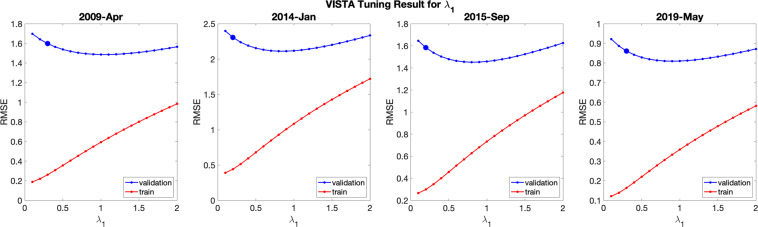
RMSE corresponding to *λ*_1_ tuning, during the tuning of which we fix ${\lambda }_{2}=(0.25,0.40,0.40,0.25)$ and ${\lambda }_{3}=(0.12,0.15,0.12,0.12)$ for the four months, respectively. Blue line shows the RMSE on the validation set and red line shows the RMSE on the training set. Colored dots show the *λ*_1_ that minimizes the average of the validation set RMSE and train set RMSE, which are the *λ*_1_ picked for each of the four months.

The best *λ*_3_ are 0.15 for 2014-Jan and 0.12 for all the other months, and so we use *λ*_3_ = 0.15 for 2012~2014 and *λ*_3_ = 0.12 for all other years. Fixing the *λ*_3_ at these optimal values, we move on and get ${\lambda }_{2}=(0.25,0.40,0.40,0.25)$ for the four year intervals. Eventually, we fix *λ*_2_ and *λ*_3_ at their optimal values, and get ${\lambda }_{1}=(0.3,0.2,0.2,0.3)$ for the four year intervals. In Fig. 8, we show the tuning results of *λ*_1_, which also shows how well the VISTA algorithm performs on a random validation set at the optimal tuning parameters chosen. We have also tried to tune the parameter in a different order: *λ*_1_ first and then *λ*_2_ and finally *λ*_3_. It turns out that tuning *λ*_3_ first gives better validation performance of the database against an independent source of TEC measurement (see Technical Validation section below), although the differences are very small.

These parameter choices would not affect the final imputation result a lot, though a more rigorous way is to decide the best hyper-parameter for every single day of data with a cross-validation step, which is definitely much more time-consuming. The tuning parameters are very similar for 2005 ~ 2011 and 2019 ~ 2020, which resonates their similarity of their geomagnetic activity levels and phase of the solar cycle. The SH order *l*_*max*_ is higher for years during 2012 ~ 2014, which reveals that the ionosphere during these years is likely more structured. The *λ*_2_ is the highest for 2015 ~ 2018 meaning that the temporal consistency is high for TEC data of these years. To summarize, we list all the tuning parameters chosen for any year within the 16-year period that our database covers in Table 3.

**Table 3:** Final tuning parameter choices for constructing the VISTA database for years in the four intervals: 2005 ~ 2011, 2012 ~ 2014, 2015 ~ 2018, 2019 ~ 2020.

Category	Notation	2005~2011	2012~2014	2015~2018	2019~2020
VISTA	*λ*_1_	0.3	0.2	0.2	0.3
	*λ*_2_	0.25	0.40	0.40	0.25
	*λ*_3_	0.12	0.15	0.12	0.12
SH	*l*_*max*_	6	9	7	7
	*v*	0.1	0.1	0.1	0.1

### Post-processing: day-to-day boundary smoothing

In order to obtain accurate TEC values, the GNSS satellite and receiver hardware biases have to be removed first. It has been shown that the satellite bias is relatively constant and is available from International GNSS Service (IGS) centers. When producing the Madrigal TEC, the receiver hardware bias is usually calculated on a daily basis^20^. Therefore, there may be very small TEC fluctuations, i.e., 1–2 TECU, across the adjacent days.

**Fig. 9:**
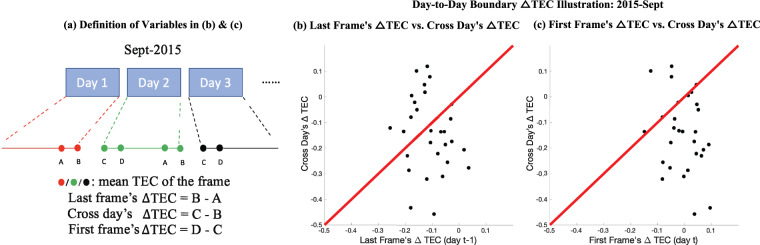
Boundary Check: Sept-2015. (**a**) shows the definition of the three quantities calculated in (**b**) and (**c**). To check if there is a “jump” of average TEC values across days, we calculate the between-frame differences of average TEC values. Each dot in (**a**) represents a frame of TEC map. Here, the “first” and “last” term mean the 1st or the 288-th frame within a day. (**b**) and (**c**) show the cross-day ΔTEC against the ΔTEC between frames belonging to the same day. The high variability of the cross-day TEC reveals that there is cross-day “jump” of TEC values and some extra smoothing is needed for the database on imputed TEC maps.

To examine if the TEC data has a boundary jump across different days, we take the data of Sept-2015, and group every two adjacent days as a single group. For each group, we calculate a few quantities near the cross-day boundary as illustrated in panel (a) of Fig. 9. More precisely, we calculate how the average TEC value has changed between frames near or cross the day-to-day boundary. In panel (b) and (c) of Fig. 9, we show the within-day TEC value inter-frame change (x-axis) against the between-day TEC value inter-frame change (y-axis) and the red line is the 45° line. One can see that typically the between-day inter-frame changes are higher (in both positive and negative directions), which are more likely due to the day-to-day TEC bias fluctuation instead of the physical dynamics of TEC values.

**Fig. 10:**
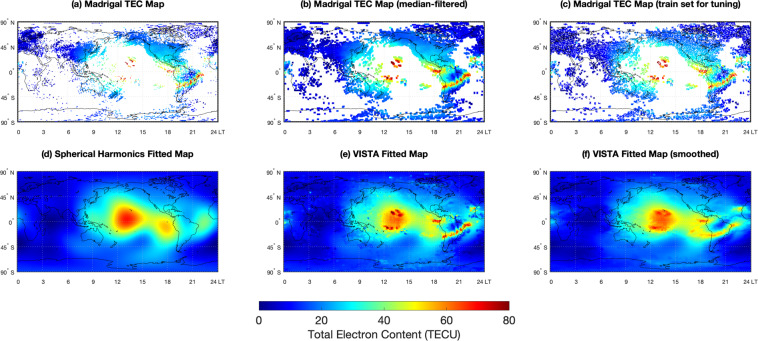
All critical TEC-related maps in our data pipeline, with the sample being the 1st frame (00:02:30 UT) of March 17, 2015. (**a**) shows the raw Madrigal TEC map after outlier removal. (**b**) shows the raw Madrigal TEC map processed by median-filter, which is the input data of our SH and VISTA algorithm. (**c**) shows the training set (~80% of the observed pixels in (**b**)) when we do parameter tuning. (**d**) is the spherical harmonics (SH) map, fitted with *l*_*max*_ = 7, *v* = 0.1 using (**b**). (**e**) shows the VISTA map using (**b**) and (**d**), with *λ*_1_ = 0.2, *λ*_2_ = 0.40, *λ*_3_ = 0.12. (**f**) shows the smoothed version of (**e**) when we apply boundary smoothing.

**Fig. 11:**
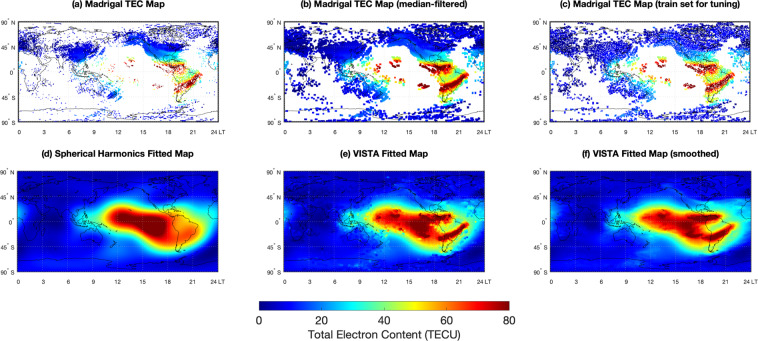
All critical TEC-related maps in our data pipeline, with the sample being the last frame (23:57:30 UT) of March 17, 2015. (**a**) shows the raw Madrigal TEC map after outlier removal. (**b**) shows the raw Madrigal TEC map processed by median-filter, which is the input data of our SH and VISTA algorithm. (**c**) shows the training set (:80% of the observed pixels in (**b**)) when we do parameter tuning. (**d**) is the spherical harmonics (SH) map, fitted with *l*_*max*_ = 7, *v* = 0.1 using (**b**). (**e**) shows the VISTA map using (**b**) and (**d**), with *λ*_1_ = 0.2, *λ*_2_ = 0.40, *λ*_3_ = 0.12. (**f**) shows the smoothed version of (**e**) when we apply boundary smoothing.

To counter this day-to-day jump of TEC levels, we pick every day’s last six frames and the first six frames of the following day, and smooth these frames with a two-sided moving average. More precisely, for every frame of the VISTA output near the cross-day boundary, ${\widehat{X}}_{t}$, we replace it with the average of its previous 6 frames: ${\widehat{X}}_{t-6},{\widehat{X}}_{t-5},\ldots ,{\widehat{X}}_{t-1}$, itself and its subsequent 6 frames: ${\widehat{X}}_{t+1},{\widehat{X}}_{t+2},\ldots ,{\widehat{X}}_{t+6}$. So the smoothed-version is ${\mathop{X}\limits^{ \sim }}_{t}=\frac{1}{13}\mathop{\sum }\limits_{k=-6}^{6}{\widehat{X}}_{t+k}$. Example of the smoothed map for March 17, 2015 is shown in (f) of Figs. 10, 11.

### Computational cost

On a single-core (i9, 2.3 GHz), 16-GB memory (2400 MHz, DDR4) CPU, a complete VISTA run (including pre-processing and SH fitting) on any day during 2005–2020 would take 5 ~ 20 minutes to fully converge. One can relax the convergence criterion a little bit (e.g. threshold changes from 10^−5^ to 10^−4^), without sacrificing any significant quality of the imputation, to cut the running time to <5 minutes.

## Data Records

The dataset is published on the Deep Blue Data system of the University of Michigan^30^, covering the years from 2005 to 2020. Each year has a separate folder containing daily data files in the format of HDF5. Each file corresponding to a single day of the year has multiple data channels as described in Table 4. The data is of 5-minute cadence, so for any daily video data there shall be 288 frames. Each frame is a 181 × 361 matrix (1°latitude × 1°longitude spatial resolution) and stored as a Numpy array using Python. All metadata, as described in Table 1, are included as headers in the HDF5 file. All channels are stored in latitude and local time grid.

**Table 4:** Description of all data channels stored in an individual data file.

Channel	Abbreviation	Description (size of data)
Spherical Harmonics	SH	Fitted spherical harmonics video. (181 × 361 × 288)
VISTA fitted	VISTA	Fitted map based on the VISTA algorithm. (181 × 361 × 288)
VISTA smoothed	VISTA_smooth	Fitted VISTA map, smoothed on the day-to-day boundary. (181 × 361 × 12)

Along with these data, the metadata described in Table 1 are also included. Each data file covers a single day during 2005 to 2020.

Here we provide a visualization of the dataset at the non-storm time 00:02:30 UT on March 17, 2015 in Fig. 10. The visualization includes the input Madrigal TEC map (after outlier removal) and three maps used in the intermediate steps of generating the final dataset and the final data based on our algorithm. (a) is the raw Madrigal TEC data. (b) is the median-filtered raw data, which is also the input data of the SH and VISTA algorithm. (c) shows the training set when we perform parameter tuning, which contains 80% of the median-filtered raw data. (d) shows the fitted spherical harmonics map with *l*_*max*_ = 7, *v* = 0.1 using (b). (e) is the output of VISTA algorithm using (b) and (d), with *λ*_1_ = 0.2, *λ*_2_ = 0.40, *λ*_3_ = 0.12. Finally, (f) shows the boundary-smoothed version of (e). Panel (e) shows the complete global TEC map with local equatorial plasma bubble signatures preserved in the postsunset sector.

Similarly, we show the plot, in the same format, for the storm time at 23:57:30 UT on March 17, 2015, which was around the peak of the geomagnetic storm based on the ring current index, in Fig. 11. One can see that the completed VISTA maps in (e) and (f) reveal the strengthened and bifurcated dayside equatorial ionization anomaly and a dearth of equatorial plasma bubble in the postsunset sector. With altered color scale, the storm-time enhanced density in the mid-latitude is also apparent. Figures 10, 11 demonstrate the capability of the VISTA algorithm under different geomagnetic activity conditions. Large-scale and rapid evolving structures, such as storm-time equatorial ionization anomaly and storm-enhanced density, are usually better captured by VISTA.

## Technical Validation

The validity of the completed TEC maps that we give in the constructed TEC database is manifested by the extensive numerical experiments shown in^17^. Through both simulated experiments and real data application, we demonstrate that the imputed TEC maps provide a reliable estimate of the global TEC, evaluated by the (test set) mean-squared error and relative-squared error.

To further validate the database using an independent source of TEC measurements, we follow the validation approach used by the IGS database^19,31^ and use the TEC measurements from the dual frequency altimeters on board the JASON satellite series as the reference TEC level. We use the JASON-1, JASON-2 and JASON-3 TEC data from the Madrigal database^18^ as the source of reference TEC data. These data are available to download with the Madrigal download API. The Madrigal JASON TEC data processing procedures are based on recommendations on the various TOPEX and JASON satellites websites^32^. Specifically, all data points with surface type or ice flags not indicating the measurement was over open water are filtered out. In addition, all points with data out of range, as defined by each satellite, are filtered out. Then 25 contiguous measurements spanning about 25 seconds are collected to calculate TEC and standard deviations. To be contiguous, two measurements must be within ten sample periods. If there is a break of ten sample periods before 25 measurements are collected, all proceeding data is dropped, and a new set of measurements is begun. When 25 contiguous measurements are acquired, the median value is then used to calculate the total electron content. Finally, the standard deviation of the 25 measurements is calculated following the conventional method.

We compare the data collected by the JASON satellites with the corresponding data in our VISTA output during 2005–2020 by converting the JASON TEC measurements into 1°latitude × 1°longitude spatial resolution with 5-minute cadence, which has the same spatio-temporal resolution as our VISTA TEC. Each JASON TEC record is assigned to its nearest neighbor in the spatio-temporal grid based on the resolution specified above. Then we calculate the difference of the TEC value measured by JASON and VISTA and group the residuals by year. We apply the same procedure to the median-filtered Madrigal TEC as well since it is the source data of VISTA. Additionally, we adjust the inter-satellite bias among the three JASON satellites based on the bias estimation in Table 5 of ^33^. Specifically, we subtract a constant of 3.5 TECu from all JASON-2 TEC measurements and 1.0 TECu from all JASON-3 measurements to make their TEC scale on par with that of JASON-1.

**Fig. 12:**
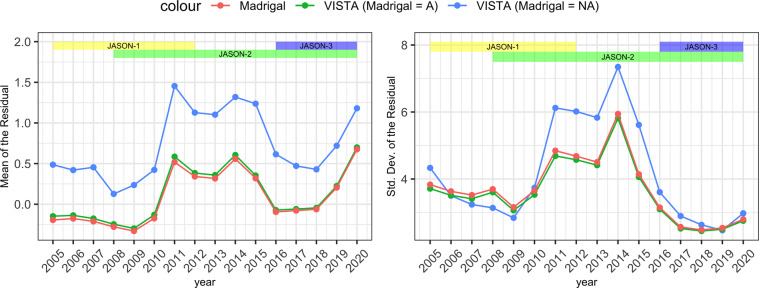
Residual mean and standard deviation, data grouped by year. Three types of data points are considered: the Madrigal TEC, the VISTA TEC with Madrigal observation (Madrigal = A) and the VISTA TEC without Madrigal observation (Madrigal = NA). The timespans that each JASON satellite provides the validation data are shown as colored bars on top. Inter-satellite biases are corrected based on [33] to make the TEC measurements from JASON-2 and JASON-3 on par with those from JASON-1. On average, each year has 10^5.8^ validation pixels.

Figure 12 shows the mean and standard deviation of the yearly residual for both the Madrigal TEC and our VISTA database. Since the VISTA algorithm typically performs better on the pixels with original observations^17^, we differentiate the pixels in the VISTA TEC based on whether the pixel has the original Madrigal TEC observation. All pixels of VISTA TEC with Madrigal TEC observations are labelled as “(Madrigal = A)”, and “(Madrigal = NA)” is used to denote the remaining pixels of VISTA TEC without the corresponding Madrigal TEC observations. One can see that the bias of the database, compared to the JASON satellite TEC measurements, show very similar trend for both the Madrigal TEC and VISTA TEC when the Madrigal data is available (i.e. Madrigal = A), and the bias gets slightly larger by 0.5 ~ 1 TECU, when no Madrigal TEC is available during the fitting. The trend is similar in the standard deviation of the residual panel. The yearly coverage of different JASON satellites is shown on top of each panel. During years 2010 ~ 2016, we see relatively higher bias and standard deviation of the residual.

Compared to the TOPEX/JASON validation results of the IGS VTEC maps^31^, the VISTA database shows lower mean and standard deviation of the bias, though the validation period does not coincide exactly. The IGS VTEC map has an average bias of 1.00 TECU and the standard deviation of the bias is around 4.42 TECU, during the validation period of 2002 ~ 2007. The VISTA database, on the other hand, show an average bias around −0.3 ~ 0.5 TECU and a standard deviation around or below 4 TECU for the period 2005 ~ 2007.

## Usage Notes

**Fig. 13:**
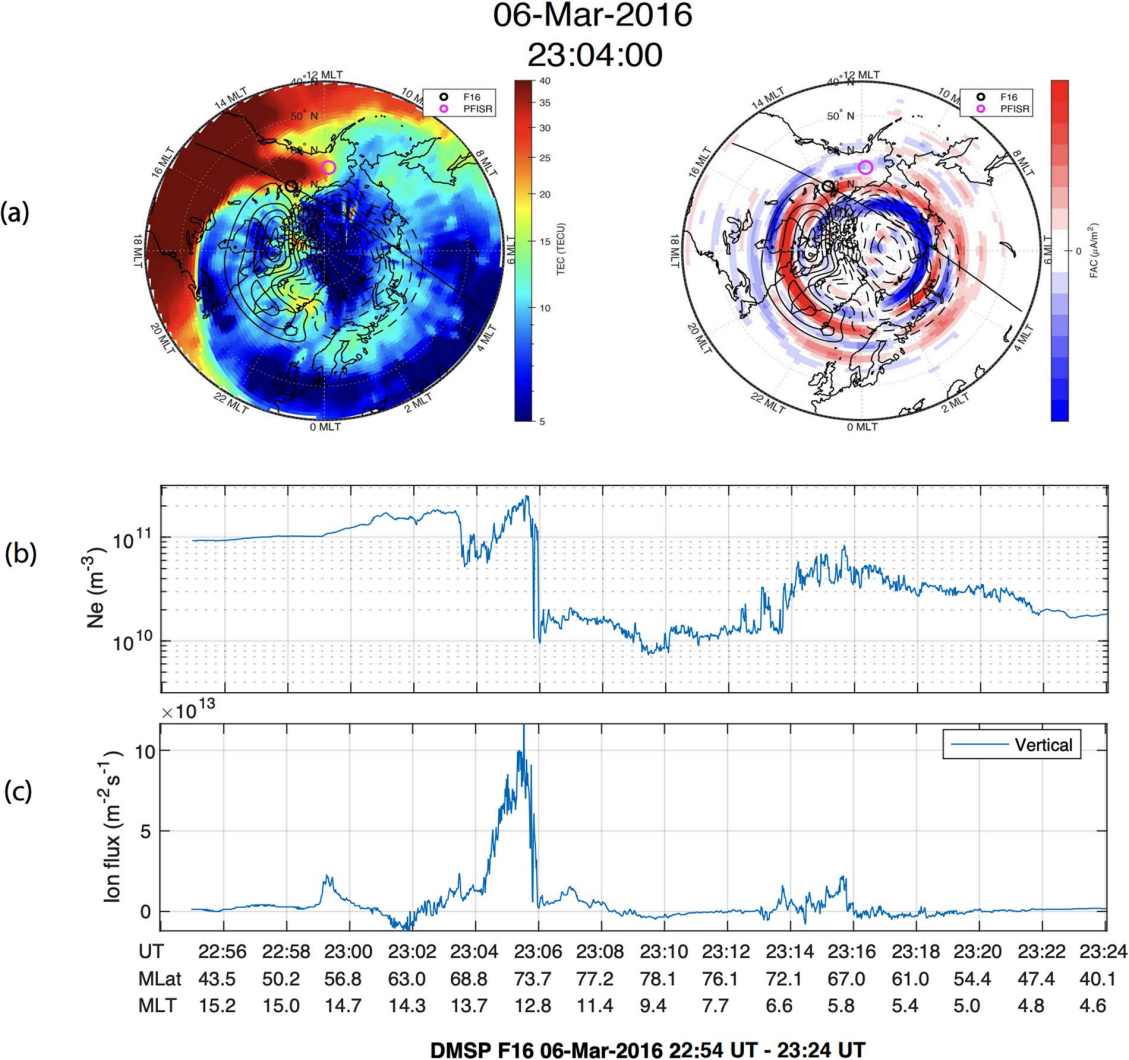
Example application of the VISTA TEC map. Large-scale TEC distribution in the Northern Hemisphere is shown in (**a**) in a polar view format. Black contours are ionospheric plasma convection pattern. (**b**) shows the ionospheric field-aligned currents together with the plasma convection pattern at the same time as the TEC map. The VISTA TEC map enabled the discovery of the high density storm-enhanced density contributions to the large ion density observed by the DMSP F16 satellite and the large ion upflow flux.

As mentioned earlier, the ionospheric TEC and its relevant products, such as ROT and ROTI, have become the most utilized parameters in the area of ionospheric research and space weather forecast. The VISTA global TEC maps with the preserved meso-scale TEC structures have tremendous potential applications in these areas. One application of the global VISTA TEC map is to provide better specification of the ionospheric high-density structures, such as storm-enhanced density and polar cap patch, and thus their role in large-scale plasma circulation and supplying ionospheric plasma to the magnetosphere via ion upflow and outflow. Recently, we^21^ took advantage of the meso-scale preservation capability of VISTA and revealed the evolution of the storm-enhanced density plume and its role in providing seed population for large ion upflow fluxes. Figure 13a shows the VISTA TEC map and field-aligned currents in the Northern Hemisphere polar region. The plume can be seen as a high TEC intrusion into the polar cap region on the dayside. The DMSP F16 satellite, labeled as a black circle, measured the elevated density associated with the plume (b) at 23:05 UT and the resulting large ion upflow fluxes (c). Without the VISTA TEC map, the interpretation of the source of the elevated density would be challenging. Other methods, such as the tomographic-kriging method in^19^, have successfully revealed tongue-of-ionizations in the polar region. Similarly, a deep learning method has reconstructed a cusp like feature during a storm^34^. The VISTA output also successfully reproduced the cusp like feature described in^34^.

## Data Availability

Details about codes that generate the dataset as well as the usage notes on accessing, downloading and pre-processing the datasets are made available on the homepage of the dataset on the Deep Blue Data system of University of Michigan^30^. Future updates of the codes and dataset will be made available on this website as well. Please contact the corresponding author for data request and questions. Additionally, our users can explore our interactive database dashboard (https://vista-tec.shinyapps.io/VISTA-Dashboard/) for more technical details and run the VISTA algorithm live.
